# Minimally Invasive Multivessel Coronary Artery Bypass Grafting Using Total Coronary Revascularization via Left Anterior Minithoracotomy in Octogenarians

**DOI:** 10.3390/jcdd12120487

**Published:** 2025-12-10

**Authors:** Christian Sellin, Marius Grossmann, Ahmed Belmenai, Margit Niethammer, Hilmar Dörge, Volodymyr Demianenko

**Affiliations:** 1 Department of Cardiothoracic Surgery, Heart-Thorax Center, Klinikum Fulda, University Medicine Marburg, Campus Fulda, 36043 Fulda, Germany; 2 Department of Cardiology, Heart-Thorax Center, Klinikum Fulda, University Medicine Marburg, Campus Fulda, 36043 Fulda, Germany

**Keywords:** minimally invasive cardiac surgery, coronary artery bypass grafting, cardiopulmonary bypass, octogenarians, MACCE, total coronary revacularization via left anterior thoracotomy (TCRAT)

## Abstract

Background: A sternum-sparing approach of minimally invasive total coronary revascularization via left anterior thoracotomy (TCRAT) demonstrated favorable early and midterm results in unselected patients with coronary artery multivessel disease. However, safety and outcomes in elderly patients remain less well defined. Particularly in octogenarians with relevant comorbidities, data are scarce, and the role of TCRAT compared to conventional coronary artery bypass grafting (CABG) remains uncertain. This study aimed to evaluate in-hospital and midterm outcomes of TCRAT in patients aged ≥ 80 years. Method: From 11/2019 to 10/2025, CABG via left anterior minithoracotomy on cardiopulmonary bypass and cardioplegic arrest was performed as a routine procedure in 859 consecutive, nonemergency patients. Among them, 82 patients (9.5%) were octogenarians, all presenting with multivessel coronary artery disease. Results: In the group of octogenarians, mean BMI was 26.5 ± 3.1 kg/m^2^, left ventricular ejection fraction was 49.2 ± 9.1% (range 20–55%), and mean EuroSCORE II was 5.1 ± 2.4. Comorbidities included diabetes mellitus (24.4%), chronic lung disease (7.3%), prior PCI (23.2%), and peripheral vascular disease (78.5%). The mean follow-up (100%) was 9.1 months. Left internal thoracic artery was used in 98.8% and radial artery was used in 43.9%. A mean of 3.0 ± 0.9 (range 2–5) anastomoses per patient was performed. Total operation time was 299 ± 64 min (range 164–480). In-hospital mortality was 1.2%, stroke rate was 1.2%, myocardial infarction rate was 0%, and repeat revascularization rate was 1.2%. At follow-up, all-cause mortality, myocardial infarction, repeat revascularization, and stroke were 4.9%, 0%, 2.4%, and 1.2%, respectively. The overall major adverse cardiac and cerebrovascular events rate (MACCE) was 7.3% at follow-up. Conclusion: TCRAT enables complete coronary artery revascularization in multivessel coronary artery disease without sternotomy and can be safely performed in octogenarians. Both in-hospital and midterm outcomes were favorable and comparable to reported contemporary outcomes of conventional CABG in elderly patients.

## 1. Introduction

Coronary artery bypass grafting (CABG) is the first-line therapy for multivessel coronary artery disease (CAD), especially in complex disease [1,2]. Therefore, CABG is the most commonly performed cardiac surgery procedure [3]. Over recent decades, life expectancy in the Western world has increased significantly, and the number of octogenarians presenting with a multivessel CAD requiring treatment is on the rise [4]. Older patients, especially those over 80 years of age, pose a particular surgical challenge. They are more likely to have comorbidities such as prior myocardial infarction, prior stroke, atrial fibrillation, heart failure, diabetes mellitus, hypertension, or kidney failure, often summarized under the term frailty [5,6]. In patients over 80 years of age, the in-hospital mortality rate is increased and major adverse cardiac and cerebrovascular events (MACCE) occur more frequently with conservative as well as interventional and surgical treatment. Additionally, other underlying diseases can complicate the treatment of coronary heart disease [7,8,9].

In 2019, a new surgical approach for complete coronary revascularization in patients with multivessel CAD via a left anterior minithoracotomy (TCRAT) was proposed and introduced [10]. Since then, the technique has been further developed [11] towards a standard method for minimally invasive CABG that avoids sternotomy, utilizing established surgical techniques [11,12,13]. Several studies have shown promising in-hospital and midterm outcomes in unselected patients [12,13,14,15,16,17] and equivalence in the rate of organ dysfunctions compared to contemporary standard CABG procedures using a full midline sternotomy [18,19]. The reduced surgical trauma by avoiding sternotomy, along with the facility of faster postoperative recovery without compromising the fundamental principles of complete coronary revascularization at the same time, may be particularly beneficial for elderly patients with limited physiological reserves. In this context, this study aimed to evaluate in-hospital and midterm outcomes of TCRAT in patients older than 80 years by analyzing clinical endpoints including major adverse cardiac and cerebrovascular events (MACCE).

## 2. Methods

### 2.1. Patient Selection and Data Collection

Between November 2019 and October 2025, a total of 859 consecutive patients underwent nonemergency isolated CABG via left anterior minithoracotomy on CPB with peripheral cannulation and cardioplegic cardiac arrest (transthoracic aortic cross-clamping) in our institution, using this technique as a default strategy in daily routine. Among them, 82 (9.5%) were octogenarians. The focus of the present study was to investigate the in-hospital and midterm outcomes of this specific subgroup. All patients were discussed in detail in the heart team [20], including a recommendation according to guideline indications [2] of which coronary arteries should be grafted. Anatomic complete revascularization was defined as the successful treatment of all significant coronary lesions with a visually estimated diameter stenosis 50% in vessels with reference vessel diameter of 1.5 mm [2]. Patients younger than 80 years of age, patients undergoing emergency procedure (i.e., same-day catheterization and operation), patients with significant atheromatous disease of the ascending aorta, patients with moderate or severe aortic regurgitation, and patients undergoing reoperation were excluded.

### 2.2. Statistical Analysis

Data were prospectively extracted from patient records and presented as mean ± standard deviation (SD) or number (percentage) and are part of our internal quality assurance documentation. The follow-up data were collected prospectively through telephone interviews, using a structured questionnaire by a study nurse, and acquisition and evaluation of medical findings. Kaplan–Meier graphs were calculated with SPSS version 29.0.0.0 (IBM-SPSS Statistics, Armonk, New York, United States).

### 2.3. Definitions of Clinical Events

Perioperative stroke was characterized as a neurological deficit attributed to an acute focal injury of the central nervous system by a vascular cause, including cerebral infarction, intracerebral hemorrhage, and subarachnoid hemorrhage. This definition is in line with the updated definition of stroke for the 21st century from the American Heart Association and American Stroke Association [21].

According to the Fourth Universal Definition of Myocardial Infarction of the Society for Cardiovascular Angiography and Interventions [22], postoperative myocardial infarction was defined as an increase in creatine kinase-MB levels within 48 h after the procedure up to 10 times the local laboratory upper limit of normal or to five times the upper limit of normal with newly occurring Q waves in 2 contiguous leads or a new persistent left bundle branch block.

MACCE were defined as a composite of all-cause mortality, myocardial infarction, repeat revascularization, and stroke.

### 2.4. Preoperative Evaluation, Anesthesia, and Surgical Technique

The preoperative evaluation, details of induction, and implementation of anesthesia and the surgical technique of TCRAT have been described by our group in detail in several recently published studies [11,12,13]. Therefore, the key points are only briefly outlined here.

A preoperative computed tomography (CT) scan, in addition to the standard institutional preoperative examinations, is crucial to screen the ascending aorta, the aortic arch, and major arterial branches, especially the iliac and femoral vessels and to detect anatomical abnormalities or atherosclerotic disease.

**Figure 1:**
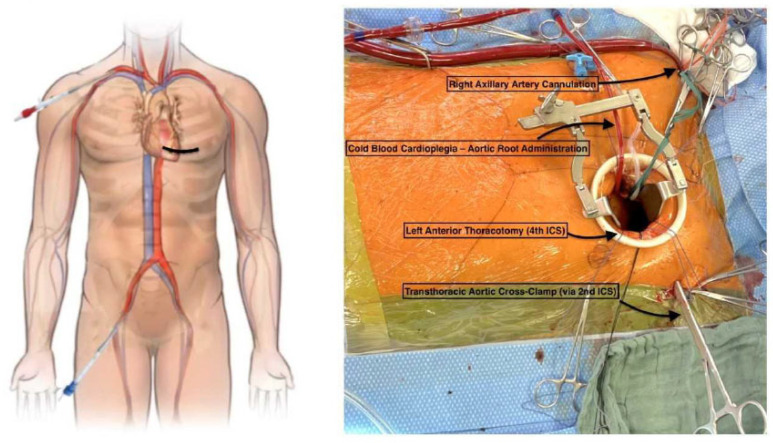
Surgical setting.

The surgical technique of TCRAT includes three key elements [10]. First, TCRAT is a sternum-sparing surgical approach by using a left-sided, anterolateral minithoracotomy at the level of the 4th intercostal space. Second, TCRAT is an on-pump technique using a peripheral cannulation strategy. This enables stable circulatory conditions, decompression and intrathoracic rotation of the heart after transthoracic aortic cross-clamp, and induction of cardioplegic cardiac arrest (Figure 1). The third key element is strategic slinging maneuvers around the great intrapericardial vessels. This is a crucial prerequisite for access to all coronary territories and enables optimal conditions for coronary artery bypass grafting, thus enabling complete anatomical revascularization.

### 2.5. Ethical Standards

This study was approved by the local ethics committee (University of Marburg, file number: 23-172 RS) and conducted in accordance with the ethical standards outlined in the 1964 Declaration of Helsinki and its subsequent amendments. The patients provided informed written consent for the publication of the study data.

## 3. Results

The study group consisted of 82 consecutive, nonemergency octogenarians (66 men; age 82.7 ± 1.8 years [range 80–88 years]) including patients with severe left ventricular dysfunction (left ventricular ejection fraction [LVEF] ≤ 30: 8.5%), prior myocardial infarction (37.8%), and patients with an elevated European System for Cardiac Operative Risk Evaluation 2 score (EuroSCORE II) above 4 (53.7%). Mean EuroSCORE II was 5.1 ± 2.4. All patients had multivessel CAD, with 41.5% having a relevant left main stem stenosis. Baseline and clinical parameters are given in Table 1.

The left internal mammary artery (LIMA) was used in 98.8%, saphenous vein graft (SVG) in 69.5%, and radial artery (RA) in 43.9%. In total, 47.6% of all patients received at least 2 arterial grafts. On average, 3.0 distal anastomoses per patient were performed, with a minimum of 2 and a maximum of 5 distal anastomoses. Left anterior descending (LAD) was grafted in 100%, the left circumflex artery (LCX) was grafted in 88%, and the right coronary artery (RCA) was grafted in 72% of all patients. Complete anatomical revascularization was achieved in 96.3%. Mean operative time was 299 ± 64 min, mean CPB time was 142 ± 38 min, and mean aortic cross clamp time was 88 ± 31 min. Operative characteristics are given in Table 2.

**Figure 2:**
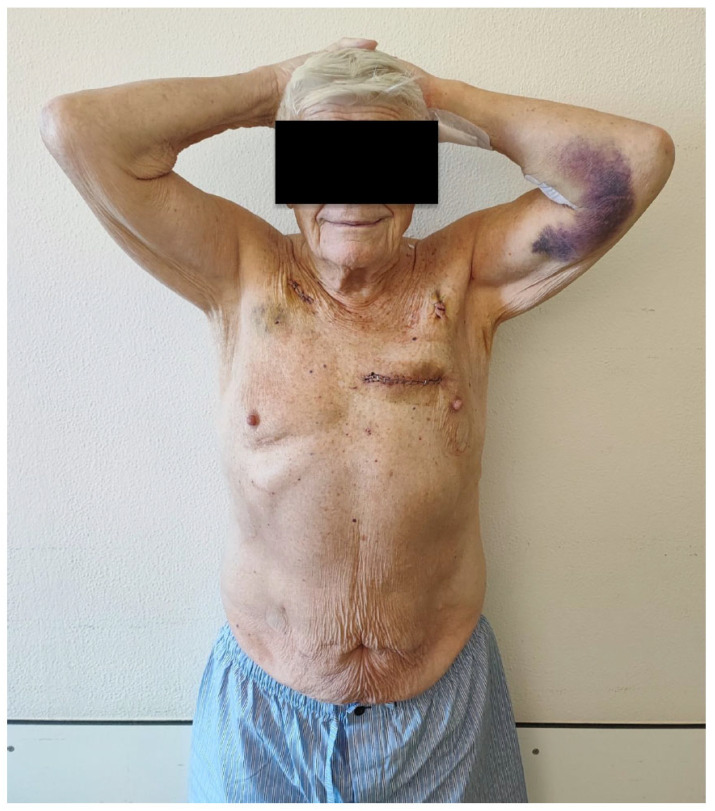
An 84-year-old patient at postoperative day 5 (LIMA to LAD, RA to LCX and PDA) elevating both arms above the head to demonstrate thoracic stability. LIMA: left internal mammary artery; LAD: left anterior descendens; RA: radial artery; LCX: left circumflex artery; PDA: posterior descending artery.

By avoiding a sternotomy, immediate mobilization was possible (Figure 2). In total, 60% of patients left the intensive care unit (ICU) within the first postoperative day. The in-hospital mortality was 1.2%. The in-hospital rate of repeated coronary artery revascularization using percutaneous coronary intervention (PCI) was 1.2%, which was a planned postoperative PCI when the posterior descending artery of the right coronary artery could not be identified intraoperatively. One patient experienced a perioperative stroke (1.2%) with minor clinical impairment. Postoperative adverse events and outcome are given in Table 3.

**Figure 3:**
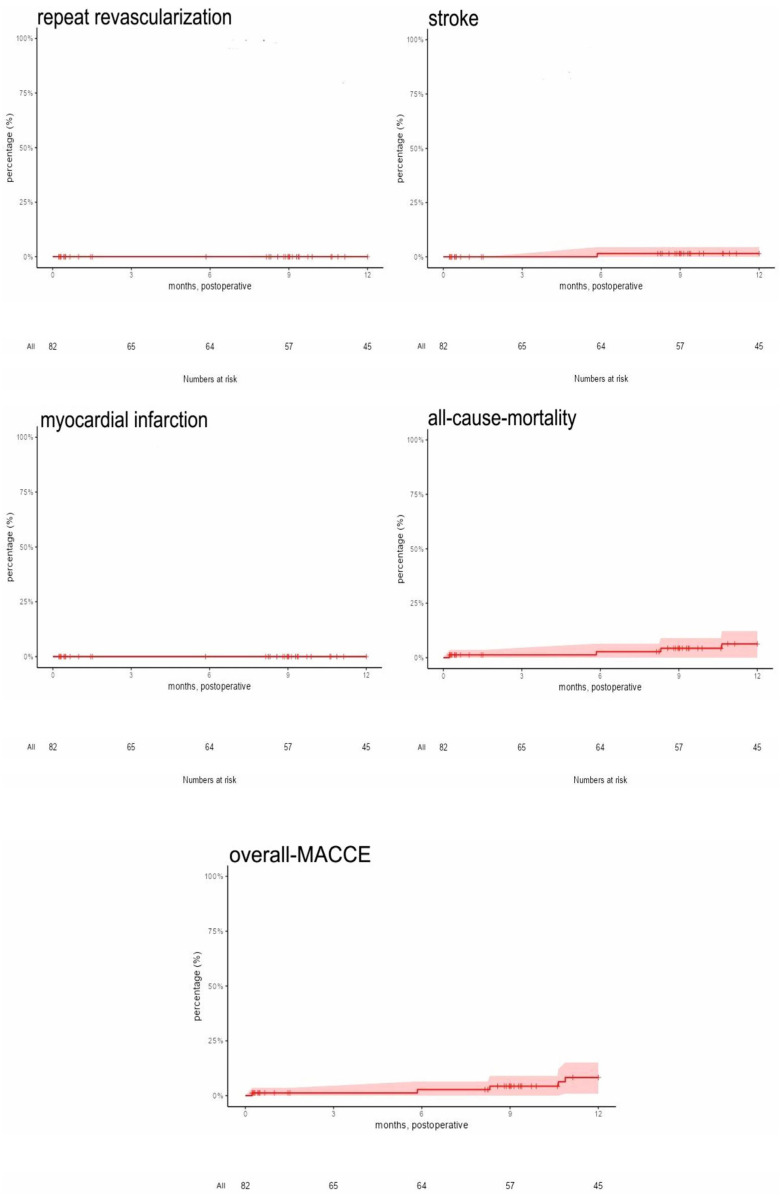
Kaplan–Meier graphs of major adverse cardiac and cerebrovascular events (MACCE) (confidence limits 95%).

Mean follow-up was 9.1 ± 4.6 months (range 0.23–12.0 months) and was completed to 100%. Median of our follow-up was 12 months (interquartile range 3.7 months). During follow-up, all-cause mortality was 4.9%, postoperative repeat revascularization was 2.4%, and postoperative stroke was found in 1.2%. No myocardial infarction was observed. Overall MACCE during the observed follow-up was 7.3%. Follow-up data are provided in Table 4 and in Figure 3.

## 4. Discussion

This is the first study that investigated the in-hospital and midterm outcomes of octogenarians after minimally invasive multivessel CABG using the TCRAT technique. This patient group is rather underrepresented in many clinical studies, but due to demographic developments in the Western world, it represents a growing and increasingly important patient collective [4,7,23,24].

Compared to important landmark studies such as the Syntax (Percutaneous Coronary Intervention versus Coronary-Artery Bypass Grafting for Severe Coronary Artery Disease) trial [25], the FAME 3 (Fractional Flow Reserve versus Angiography for Multivessel Evaluation) trial [26], and the first and so far only published study on in-hospital and midterm results using the TCRAT technique, the present study shows an increased operative risk in the group of octogenarians when analyzing baseline characteristics. This is reflected by a mean EuroSCORE II of 5.1 and a proportion of over 50% of patients with a EuroSCORE II greater than 4. A recently published meta-analysis of CABG and PCI in octogenarians with left main or multivessel CAD reported comparable baseline characteristics to our data [24].

Substantial benefit from surgical compared to interventional coronary revascularization is most pronounced in patients with multivessel CAD, diabetes mellitus, and with reduced LVEF [2]. Added to this, CABG patients often experience high comorbidity and surgical risk—this applies in particular to the group of octogenarians. In the present study, we included octogenarians with diffuse multivessel CAD requiring complex coronary surgery, with very low LVEF, obesity, and chronic obstructive pulmonary disease and with high surgical risk.

All coronary arteries with a diameter exceeding 1.5 mm and a luminal reduction of 50% or more should be grafted [2] to achieve a complete anatomical revascularization. According to this definition, complete anatomical revascularization was accomplished in 96.3% of patients in our study. The number of grafts performed in patients with multivessel CAD often is considered as an indirect parameter for the completeness of revascularization [27]. The average number of grafts performed in the present study was 3.0, which compares well to the average number of 3.0 grafts reported for conventional CABG in the German Heart Surgery Report 2023 [28] and is consistent with the data from the only study published to date on midterm results after minimally invasive CABG using the TCRAT technique in an all-comer population [13]. According to recent revascularization guidelines [2], we achieved a multi-arterial graft configuration in more than 47%, despite the advanced patient age.

In our study, the in-hospital mortality rate was 1.2%, which compares favorably with the mortality rate of 2.1% reported by several studies in current CABG patients overall and the reported 30-day mortality rate of 4% to 16.8% in the octogenarian group [9,29,30,31].

The stroke rate for CABG in octogenarians is reported to be between 1.3% for off-pump procedures and 2.4% for on-pump procedures [32]. However, the data are inconsistent. A recently published multicenter study showed no difference in stroke rates between on-pump and off-pump procedures in this specific group of patients [9]. The same applies to the perioperative rate of acute renal failure, postoperative dialysis, or sepsis. In the present study, we found a perioperative stroke rate of 1.2%, which is comparable to the reported off-pump results and lower than the reported results for on-pump procedures. A possible explanation for this low stroke rate could be the peripheral arterial cannulation strategy via the right axillary artery [33], which avoids retrograde flow through the aortic arch, minimizing the potential for retrograde embolization from a diseased aorta combined with a preoperative CT scan. In summary, the present study shows an in-hospital MACCE rate of 3.7%. This is exceptionally low compared to reported data of 10–13% [4,34].

The reported 1-year follow-up results of the groundbreaking myocardial revascularization trials from recent decades [35], like the SYNTAX [25] and the FAME 3 [26] trial, are in line with the findings in our study. However, our study included significantly older patients with a higher surgical risk profile. The SYNTAX trial reported a 1 year follow-up MACCE rate between 12.9% and 15.5%, depending on the SYNTAX score. Compared to that, the FAME 3 trial reported a MACCE rate of 6.9% after 1 year. The observed MACCE rate of 7.3% after a mean follow-up period of 9.1 months is favorable and competitive compared to the trials mentioned above of conventional bypass surgery using full midline sternotomy. The DuraGraft Registry [36], a recently published multicenter registry study, prospectively examined the clinical outcome after contemporary conventional CABG and reported a MACCE rate of 7.8% after 1 year. These results are consistent with our findings, despite the increased risk profile in our patient population. Comparing the results of our follow-up with the results of studies focusing on octogenarians, an increased 1-year mortality between 6.7% and 10.5% and a MACCE rate of 14.5% are reported [8,34].

Based on our findings, minimally invasive sternum-sparing CABG using the TCRAT technique emerges as a robust and safe option for the treatment of multivessel CAD that warrant its active consideration, especially in octogenarians. The decision to operate should take into account the patient’s overall condition, mobility, and comorbidities. Age plays an important, but not the only, role.

### Study Limitations

There are several limitations of our study.

The present study is a prospective, single-center study using a single-arm design to investigate a recently introduced surgical approach and its results in a special group of patients. There is a certain patient selection because octogenarians who showed severe calcifications of the ascending aorta or need for emergency surgery were not included in the present study. The number of patients studied is relatively small. Larger patient numbers could allow for more precise and meaningful analyses. Furthermore, mean follow-up of 9.1 months was only midterm. Longer follow-up periods are necessary to further assess the role of TCRAT in this particular patient collective.

Despite all limitations, the results of the present study are relevant to further advance the development of treatment processes for multivessel CAD in older populations.

In conclusion, TCRAT enables complete coronary artery revascularization in multivessel CAD without sternotomy and can be safely performed in octogenarians. Both in-hospital and midterm results were favorable and comparable to contemporary outcomes of conventional CABG in elderly patients.

## Figures and Tables

**Table 1:** Baseline and clinical parameters.

Variables	*n* = 82 *n* (%)
Age (years)	82.7 ± 1.8 (80–88)
Male	66 (80.5%)
BMI (kg/m^2^)	26.5 ± 3.1 (20.1–35.4)
Hypertension	80 (97.6%)
Diabetes mellitus	20 (24.4%)
Chronic lung disease	6 (7.3%)
Current smoker	5 (6.1%)
Peripheral or cerebrovascular arterial disease	64 (78.5%)
Creatinine (mg/dL)	1.2 ± 0.6 (0.7–6)
EuroSCORE II (%) EuroSCORE II ≥ 4	5.1 ± 2.4 (1.5–17.5) 44 (53.7%)
LVEF (%) LVEF ≤ 30	49.2 ± 9.1 (20–55) 7 (8.5%)
2-vessel disease	14 (17.1%)
3-vessel disease	68 (82.9%)
Left main stenosis > 50%	34 (41.5%)
Prior NSTEMI	31 (37.8%)
Prior PCI	19 (23.2%)

Data are expressed as mean ± SD or absolute values (percentage%). Minimum–maximum values are in parenthesis. BMI: body mass index; LVEF: left ventricular ejection fraction; NSTEMI: non-ST-elevation myocardial infarction; PCI: percutaneous coronary intervention.

**Table 2:** Operative characteristics.

Variables	*n* = 82 *n* (%)
Aortic cross-clamp time, min Median IQR	88 ± 31 (22–162) 89 44
CPB time, min Median IQR	142 ± 38 (56–243) 142 49
Operative time, min Median IQR	299 ± 64 (164–480) 301 143
Number of distal anastomoses	3.0 ± 0.9 (2–5)
Conduits LIMA RA SVG	81 (98.8%) 36 (43.9%) 57 (69.5%)
Complete arterial revascularization	25 (30.5%)
Multiple arterial revascularization	39 (47.6%)
Complete anatomical revascularization	78 (95.1%)
Cannulation-related events (bleeding or intraoperative dissection)	0 (0.0%)

Data are presented as mean (±standard deviations) or absolute values (percentage%). CPB: cardiopulmonary bypass; LIMA: left internal mammary artery; RA: radial artery; SVG: saphenous vein graft; min: minutes; IQR: interquartile range.

**Table 3:** In-hospital outcome.

Variables	*n* = 82 *n* (%)
Rethoracotomy	6 (7.3%)
ARF requiring dialysis	3 (3.7%)
Pneumonia	1 (1.2%)
New onset of atrial fibrillation	7 (8.5%)
ICU length of stay, days Median IQR	2.7 ± 4.8 (1–37) 1 1
In-hospital length of stay, days Median IQR	13.0 ± 10.9 (6–71) 9 5
Stroke	1 (1.2%)
Myocardial infarction	0 (0.0%)
Postoperative PCI Of these planned procedures	1 (1.2%) 1 (1.2%)
In-hospital mortality	1 (1.2%)
In-hospital MACCE	3 (3.7%)

Data are presented as mean ± SD or absolute values (percentage%). ARF: acute renal failure; ICU: intensive care unit; min: minutes; IQR: interquartile range; PCI: percutaneous coronary intervention; MACCE: major adverse cardiac and cerebrovascular events.

**Table 4:** Midterm follow-up data.

Variables	*n* = 82 *n* (%)
Stroke	1 (1.2%)
Myocardial infarction	0 (0.0%)
Postoperative PCI	2 (2.4%)
Cardiac mortality	1 (1.2%)
All-cause mortality Cardiac death Non-cardiac death	4 (4.9%) 0 (0.0%) 4 (4.9%)
Overall MACCE	6 (7.3%)

Data are presented as absolute values (percentage%). PCI: percutaneous coronary intervention; MACCE: major adverse cardiac and cerebrovascular events.

## Data Availability

The data presented in this study are available on request from the corresponding author.

## Abbreviations

The following abbreviations are used in this manuscript:

CABG	coronary artery bypass grafting
CAD	coronary artery disease
CPB	cardiopulmonary bypass
CT	computed tomography
ICU	intensive care unit
LAD	left anterior descending
LCX	left circumflex artery
LIMA	left internal mammary artery
LVEF	left ventricular ejection function
MACCE	major adverse cardiac and cerebrovascular events
PCI	percutaneous coronary intervention
RA	radial artery
RCA	right coronary artery
SD	standard deviation
SVG	saphenous vein graft
TCRAT	total coronary revascularization via left anterior thoracotomy
